# Epigenome overlap measure (EPOM) for comparing tissue/cell types based on chromatin states

**DOI:** 10.1186/s12864-015-2303-9

**Published:** 2016-01-11

**Authors:** Wei Vivian Li, Zahra S. Razaee, Jingyi Jessica Li

**Affiliations:** Department of Statistics, 8125 Math Sciences Bldg., University of California, Los Angeles, CA, 90095-1554 USA; Department of Human Genetics, University of California, Los Angeles, CA, 90095-7088 USA

**Keywords:** Comparative epigenomics, Tissue and cell type characteristics, Chromatin states, Enhancers and promoters, Histone modification, Disease variants, Multiple testing

## Abstract

**Background:**

The dynamics of epigenomic marks in their relevant chromatin states regulate distinct gene expression patterns, biological functions and phenotypic variations in biological processes. The availability of high-throughput epigenomic data generated by next-generation sequencing technologies allows a data-driven approach to evaluate the similarities and differences of diverse tissue and cell types in terms of epigenomic features. While ChromImpute has allowed for the imputation of large-scale epigenomic information to yield more robust data to capture meaningful relationships between biological samples, widely used methods such as hierarchical clustering and correlation analysis cannot adequately utilize epigenomic data to accurately reveal the distinction and grouping of different tissue and cell types.

**Methods:**

We utilize a three-step testing procedure–ANOVA, t test and overlap test to identify tissue/cell-type- associated enhancers and promoters and to calculate a newly defined Epigenomic Overlap Measure (EPOM). EPOM results in a clear correspondence map of biological samples from different tissue and cell types through comparison of epigenomic marks evaluated in their relevant chromatin states.

**Results:**

Correspondence maps by EPOM show strong capability in distinguishing and grouping different tissue and cell types and reveal biologically meaningful similarities between Heart and Muscle, Blood & T-cell and HSC & B-cell, Brain and Neurosphere, etc. The gene ontology enrichment analysis both supports and explains the discoveries made by EPOM and suggests that the associated enhancers and promoters demonstrate distinguishable functions across tissue and cell types. Moreover, the tissue/cell-type-associated enhancers and promoters show enrichment in the disease-related SNPs that are also associated with the corresponding tissue or cell types. This agreement suggests the potential of identifying causal genetic variants relevant to cell-type-specific diseases from our identified associated enhancers and promoters.

**Conclusions:**

The proposed EPOM measure demonstrates superior capability in grouping and finding a clear correspondence map of biological samples from different tissue and cell types. The identified associated enhancers and promoters provide a comprehensive catalog to study distinct biological processes and disease variants in different tissue and cell types. Our results also find that the associated promoters exhibit more cell-type-specific functions than the associated enhancers do, suggesting that the non-associated promoters have more housekeeping functions than the non-associated enhancers.

**Electronic supplementary material:**

The online version of this article (doi:10.1186/s12864-015-2303-9) contains supplementary material, which is available to authorized users.

## Background

While all human tissue and cell types largely preserve the biological information in the DNA sequence of the human genome, the epigenomic landscapes of different tissue and cell types vary considerably, resulting in distinct gene expression programs, biological functions and phenotypic variations. Epigenomic information, such as DNA methylation, covalent histone modifications and DNA accessibility in each tissue and cell type can be investigated using high-throughput sequencing technologies such as Bisulfite-seq, ChIP-seq and DNase-seq [1]. The genome-wide dynamics of epigenomic marks in their relevant chromatin states are considered to bridge genotypes and phenotypes, and they can promote the discovery of biologically meaningful relationships between vast cell types, tissues and lineages [2–4].

Previous research mostly relied on gene expression profiles to study the relationships of samples from different tissue and cell types [5, 6]. The 111 reference epigenomes from the NIH Roadmap Epigenomics Program [4] together with the 16 epigenomes reported by the ENCODE project [7] provided a global view of the epigenomic information covering a large variety of human tissue and cell types. ChromHMM utilized them to build a genome-wide annotation of chromatin states [8]. These large-scale datasets enabled us to study the relationships among tissue and cell types from a new perspective: the similarity of tissue and cell types in terms of histone modification marks evaluated in relevant chromatin states.

Histone modifications at enhancers and promoters in the human genome were found to both reflect and explain global cell-type-specific gene expression [7, 9]. Kundaje et al. showed that pairwise similarity matrices of diverse histone marks could be used to distinguish different subsets of the samples [2]. The similarity matrices were pairwise Pearson correlation values separately calculated for a variety of epigenomic marks. In the same work, they also performed hierarchical clustering of the 111 reference epigenomes using H3K4me1 signal in enhancers (identified by a 15-state HMM model) and showed consistent grouping of biologically similar cell and tissue types, including ES cells, iPS cells, T cells, B cells, adult brain, fetal brain, digestive systems, smooth muscle and heart. Heintzman et al. performed *k*-means clustering on chromatin modifications from both promoters and enhancers [9]. Their results suggested that the chromatin states at promoters are largely invariant across different cell types. In contrast, enhancers reveal cell-type-specificity in clustering and correlate to cell-type-specific gene expression programs on a global scale.

The recent large-scale imputation of epigenomic datasets provided a more consistent and robust resource for capturing sample relationships and dynamic epigenomic information across cell types [10]. Ernst et al. found that compared with the original data, the imputed data led to a correlation matrix of epigenomic features with a more strongly pronounced block structure, suggesting that the imputed data provided a stronger basis for clustering samples into their true tissue or cell type.

Despite the fact that hierarchical clustering and correlation analysis have been shown useful in studying the relationships of biological samples across tissue and cell types, there are many limitations in their use. In the tree representation of hierarchical clustering, it is often difficult to identify the number of groups. In correlation analysis, both Pearson and Spearman correlation coefficients usually provide a noisy correlation matrix of samples, making the detection of sample groups another challenge. Therefore, in order to find a clear correspondence map and distinct grouping of samples based on epigenomic features, we need new methods. Here we propose a new measure–Epigenome Overlap Measure (EPOM)–to distinguish different tissue and cell groups by performing a three-step testing procedure on large-scale epigenomic datasets.

## Methods

**Fig. 1:**
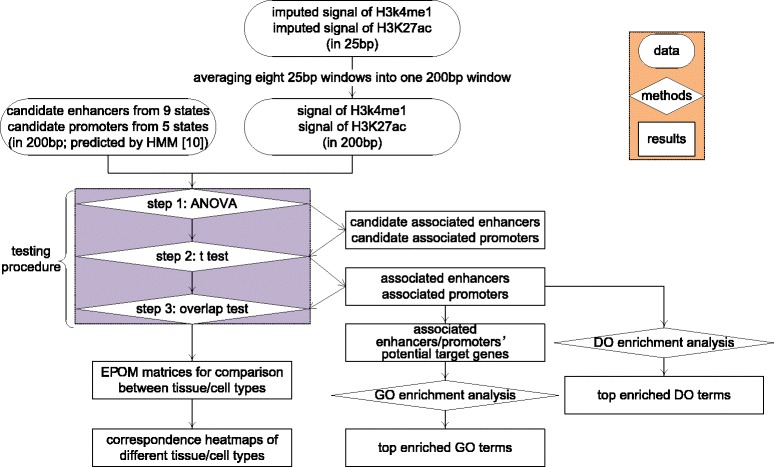
Outline of EPOM. The orange box gives the legend of the outline: the rounded rectangles represent data taken as input of EPOM; the diamonds represent methods and analysis used in our work; the rectangles represent results and outcomes of our methods. More details of the testing procedure are given in Fig. 2

We describe our method in the following three subsections. In the first subsection, we introduce how the chromatin states are defined; in the second subsection, we describe how we select the histone marks (HMs) based on their relationships with the chromatin states of interest; in the third subsection, we introduce our main three-step testing procedure. The outline of our method is illustrated in Fig. 1.

**Fig. 2:**
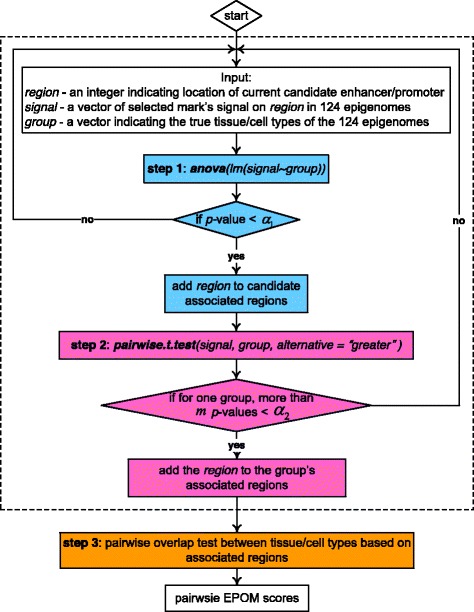
Diagram of EPOM’s three-step testing procedure

### Selection of chromatin states

We study and evaluate the capacity of our method in mapping and grouping different tissue and cell types using histone marks at both enhancer and promoter regions.

In our study, we use chromatin states of genome-wide 200 bp regions learned by a 25-state multivariate Hidden Markov Model (HMM) [10]. The chromatin states were learned from the imputed data of 12 marks: H3K4me1, H3K4me2, H3K4me3, H3K9ac, H3K27ac, H4K20me1, H3K79me2, H3K36me3, H3K9me3, H3K27me3, H2A.Z, and DNase, across 127 human tissue and cell types (111 from Roadmap and 16 from ENCODE) [2, 10]. Ernst and Kellis [10] interpreted biological meanings of each state through computing the overlap and neighborhood enrichments of the state with respect to various types of functional annotations such as CpG islands, exons, genes, etc. We consider their identified enhancer-related and promoter-related chromatin states (Table 1) as our candidate enhancers and candidate promoters respectively, with a length of 200 bp for each candidate enhancer (or promoter) region. Description of these chromatin states is summarized in Table 1.

**Table 1:** Chromatin state description

Candidate promoters		Candidate enhancers	
State number	Description	State number	Description
1	Active TSS	10	Transcription 5’ enhancer
2	Promoter upstream TSS	11	Transcription 3’ enhancer
3	Promoter downstream TSS with DNase	12	Transcription weak enhancer
4	Promoter downstream TSS	13	Active enhancer 1
22	Poised promoter	14	Active enhancer 2
23	Bivalent promoter	15	Active enhancer flank
		16	Weak enhancer 1
		17	Weak enhancer 2
		18	Enhancer acetylation only

Note: The state numbers and the description are from [10]. The state numbers were designated by the 25-state-HMM. The description was based on the enrichment of functional annotations and experimentally observed characteristics

### Selection of histone modification marks

H3 lysine 4 monomethylation (H3K4me1) was observed to distribute in a cell-type-specific manner and associate with enhancer regions: predicted enhancers showed H3K4me1 enrichment [9, 11]. It was also verified that candidate enhancer states all shared higher frequencies of H3K4me1 than other methylation marks [12]. Another histone modification mark, H3 acetyl K27 (H3K27ac) was associated with active promoters in mammalian cells [13] and predicted enhancers [9]. Hence, we examined the signals of H3K4me1 and H3K27ac in the candidate enhancer (or promoter) regions and attempted to identify the regions where the signals can distinguish different tissue/cell types (we also extended our method to include a third mark H3K4me3 and the results are in Additional file 1). The signals of each mark are − log10 transformed *p*-values, which represent the enrichment of ChIP-seq read counts based on a Poisson distribution. A stronger signal represents a more statistically significant enrichment of histone modification [2]. The original signals are at 25 bp resolution. We compressed the signals into 200 bp resolution by taking the average of every eight 25 bp windows, so that the signals and our candidate enhancer and promoter regions can be perfectly aligned as 200 bp windows.

### Testing procedure

Given the signals of H3K4me1 and H3K27ac on 124 reference epigenomes divided into 16 tissue and cell types (we excluded the three tissue and cell types that only contain one sample) and the locations of candidate enhancers and candidate promoters, we used a three-step testing procedure (please see Fig. 2) to calculate pairwise EPOM scores and study the relationships among different tissue and cell types. The 16 tissue and cell type groups are: embryonic stem cells (ESC), induced pluripotent stem cells (iPSC), ESC-derived cells (ES-deriv.), Blood & T-cells, HSC & B-cells, Mesenchymal stem cells (Mesench.), Epithelial, Neurosphere (Neurosph.), Thymus, Brain, Muscle, Heart, Smooth Muscle (Sm. Muscle), Digestive, Other, and ENCODE cell lines (ENCODE2012).

- We use ANOVA to test whether a histone mark has the same group mean signals across the 16 tissue and cell types. We denote the group mean signal of the mark at the *k*-th candidate enhancer (or promoter) in the *i*-th tissue/cell type as *μ*_*i,k*_. Then the *k*th null hypothesis can be expressed as
  (1) $$ H_{0,k}: \mu_{1,k}=\mu_{2,k}=\cdots=\mu_{16,k}  $$
  We apply a threshold *α*_1_ to the resulting Bonferroni-corrected *p*-values and refer to region *k* as a *candidate associated enhancer (or promoter)* if the null hypothesis *H*_0,*k*_ is rejected.
- We use one-tailed t-test to perform pairwise comparison between the 16 tissue/cell types, so as to identify the associated enhancers (or promoters) of each tissue/cell type. Given two different tissue/cell types *i* and *j* and the *k*-th candidate associated enhancer (or promoter), the null hypothesis is
  (2) $$ H_{0,ijk}: \mu_{i,k}\leq\mu_{j,k}  $$
  We apply a threshold *α*_2_ to the resulting *p*-values and we consider the signal of tissue/cell type *i* to be significantly higher than that of tissue/cell type *j* on region *k* if the null hypothesis *H*_0,*ijk*_ is rejected. For the *i*-th tissue/cell type, if *H*_0,*ijk*_ is rejected for more than *m* times among all *j*≠*i*, we define region *k* as an *associated enhancer (or promoter)* of tissue/cell type *i*. We separately identify the H3K4me1-based and H3K27ac-based associated enhancers and promoters of each tissue/cell type. Then we combine the information of the two histone marks by taking the union of their associated enhancers (or promoters). That is, for each tissue/cell type we take the union of the two marks’ associated enhancers (or promoters) and use the union as the associated enhancers (or promoters) of that tissue/cell type.
- We perform the overlap test, described in next subsection, on the discovered associated enhancers (promoters) to calculate EPOM scores between every pair of tissue/cell types.

In this paper, we set the thresholds as *α*_1_=10^−10^, *α*_2_=0.01, and *m*=13 or 14. In our testing procedure, the ANOVA procedure in Step 1 aims to filter out the candidate enhancer (or promoter) regions whose HM signals do not have significant variations across all biological conditions (i.e., tissue and cell types). Step 2 consists of pairwise two-sample t-tests, which aim to find associated regions for each biological condition, such that these regions’ HM signals in this condition are significantly higher than in at least *m* other conditions. Steps 1 and 2 are not redundant but complementary to each other. Step 1 can largely reduce the number of candidate associated regions to be tested in Step 2, so that Step 2 will find the associated regions that not only have high signals in one biological condition but also have strong signal variations across conditions. In addition, Step 1 can largely reduce computational time in Step 2, so as to increase the computational efficiency of the EPOM method. Step 2 is necessary to identify associated regions that carry cell-type-specific characteristics, because it centers on each biological condition in its search for associated regions. The two steps together ensure that the identified associated regions have strong differentiating capability of biological conditions and thus serve as good candidates for the overlap test in Step 3.

### Overlap test in the three-step testing procedure

The overlap test, a procedure to check the significance of the overlap of two samples, has been demonstrated as a powerful procedure to map developmental stages from the same or different species based on transcriptomic data [14]. Here we use it to calculate pairwise EPOM scores. Given two tissue/cell types, we compare them by testing the overlap of their associated enhancers (or promoters), e.g., genomic region sets *A* and *B*. We consider the union of the associated enhancers (or promoters) of all tissue/cell types after step 2 as the population and *A* and *B* as two samples drawn from the population. The null hypothesis is that *A* and *B* are two independent samples, while the alternative hypothesis is that *A* and *B* are dependent samples. The test statistic is the number of regions shared by *A* and *B*. Given *n* (the population size), |*A*| and |*B*| (the sizes of *A* and *B*), the larger the test statistics is, the higher the likelihood that the null hypothesis will be rejected. The *p*-value of the test statistic is calculated as:

(3) $$ p = \sum_{i = |A\cap B|}^{\min(|A|,|B|)} \frac{{n \choose i}{n-i \choose |A|-i} {n-|A| \choose |B|-i}}{{n \choose |A|}{n \choose |B|}}  $$

Then we define the EPOM score between samples *A* and *B* as

(4) $$ \text{EPOM score} = -\log_{10}(\text{Bonferroni-corrected}\ p)  $$

The larger the EPOM score is, the more likely that *A* and *B* are dependent and the more epigenomic characteristics they share, and vice versa.

## Results

From the chromatin states discovered by the 25-state HMM model [10], we identified 4,056,578 candidate enhancers and 1,401,636 candidate promoters (both in 200-bp units) from human Chromosomes 1–22 and Chromosome X. In Step 1 (ANOVA) of the testing procedure, we reduced our target regions to 1,646,842 candidate associated enhancers and 533,086 candidate associated promoters using H3K4me1, and 834,975 enhancers and 306,062 promoters using H3K27ac. Then after Step 2 (t test) of the testing procedure, the numbers of associated regions discovered for different tissue and cell types are summarized in Table 2. On one hand, our study verified that large proportion of promoters are housekeeping, which was consistent with the observation via *k*-means clustering and Pearson correlation that promoter regions are largely invariant across different tissue/cell types [9]. On the other hand, we did not observe the association proportion (number of associated regions divided by number of candidate regions) of promoters to be necessarily lower than the association proportion of enhancers. In ten tissue and cell types: ESC, iPSC, ES-deriv., Blood & T-cell, Mesench., Thymus, Brain, Sm. Muscle, Digestive and Other, enhancers’ association proportions are about 1.1-1.9 times of promoters’; in the other six tissue and cell types: HSC & B-cell, Muscle, Epithelial, Neurosphere, Heart and ENCODE2012, enhancers’ association proportions are only about 50 %–90 % of promoters’.

**Table 2:** Numbers and proportions of enhancer/promoter regions associated with various tissue/cell types

	H3K4me1		H3K27ac		Union of the two HMs	
Tissue/cell type	Enhancers	Promoters	Enhancers	Promoters	Enhancers	Promoters
Numbers of associated regions						
ESC	43,459	8,852	13,981	4,352	51,666	11,942
iPSC	9,770	1,814	12,079	2,050	20,553	3,697
ES-deriv.	2,242	544	330	79	2,458	598
Blood & T-cell	25,8638	58,525	113,189	35,189	272,139	74,705
HSC & B-cell	29,013	19,855	12,847	9,538	37,371	26,889
Mesench.	242,345	61,975	178,065	64,313	302,647	94,113
Epithelial	4,118	1,243	481	386	4,463	1,614
Neurosph.	13,363	4,614	9,046	4,084	19,202	7,703
Thymus	10,724	2,194	11,158	2,076	18,217	3,800
Brain	152,652	22,362	149,195	30,917	209,745	40,745
Muscle	15,288	3,496	13,166	5,020	23,513	7,416
Heart	3,225	1,098	8,601	3,219	10,458	3,951
Sm. Muscle	38,548	5,386	32,615	6,257	49,460	9,107
Digestive	25,782	4,980	5,528	2,242	28,186	6,563
Other	5	0	0	1	5	1
ENCODE2012	25	13	333	18	55	30
% of associated regions among candidate regions						
ESC	1.07	0.63	0.34	0.31	1.27	0.85
iPSC	0.24	0.13	0.30	0.15	0.51	0.26
ES-deriv.	0.06	0.04	0.01	0.01	0.06	0.04
Blood & T-cell	6.38	4.18	2.79	2.51	6.71	5.33
HSC & B-cell	0.72	1.42	0.32	0.68	0.92	1.92
Mesench.	5.97	4.42	4.39	4.59	7.46	6.71
Epithelial	0.10	0.09	0.01	0.03	0.11	0.12
Neurosph.	0.33	0.33	0.22	0.29	0.47	0.55
Thymus	0.26	0.16	0.28	0.15	0.45	0.27
Brain	3.76	1.60	3.68	2.21	5.17	2.91
Muscle	0.38	0.25	0.32	0.36	0.58	0.53
Heart	0.08	0.08	0.21	0.23	0.26	0.28
Sm. Muscle	0.95	0.38	0.80	0.45	1.22	0.65
Digestive	0.64	0.36	0.14	0.16	0.69	0.47
Other	0.00	0.00	0.00	0.00	0.00	0.00
ENCODE2012	0.00	0.00	0.01	0.00	0.00	0.00

### EPOM between different tissue/cell types

**Fig. 3:**
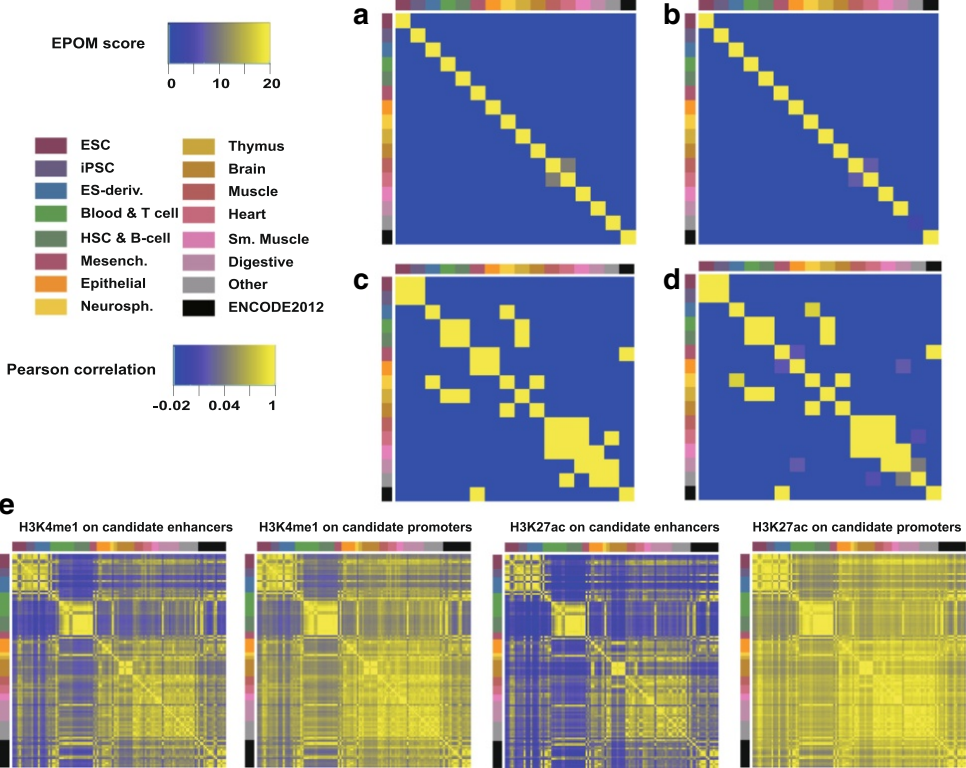
**a**–**d** Correspondence maps of EPOM scores saturated at 20 (all the values larger than 20 are set to 20). **a** Calculated based on associated enhancers (threshold *m*=14 in t test). **b** Calculated based on associated promoters (threshold *m*=14 in t test). **c** Calculated based on associated enhancers (threshold *m*=13 in t test). **d** Calculated based on associated promoters (threshold *m*=13 in t test). **e** Correspondence maps of Pearson correlation coefficients calculated from H3K4me1 and H3K27ac signals in candidate enhancers and candidate promoters. Axis colors mark different true tissue/cell types

We summarize the EPOM scores of all pairwise comparisons based on the identified associated enhancers and associated promoters respectively. The two resulting matrices were plotted as heatmaps to illustrate the correspondence maps of epigenomes, as shown in Fig. 3a, b. The two heatmaps based on the associated enhancers and the associated promoters are highly consistent, showing a clear diagonal pattern corresponding to the biological groupings of tissue and cell types. The only off-diagonal element is a weak mapping between Muscle and Heart. This is not surprising since heart consists mostly of cardiac muscle cells. Figure 3e and Additional file 2 illustrate the correlation matrices based on H3K4me1 and H3K27ac signals at the candidate enhancer and promoter regions before and after our Step 1 ANOVA, respectively. These heatmaps from correlation analysis can only roughly distinguish three large groups of human tissue and cell types. Comparing the correspondence maps established by EPOM and correlation analysis, we can see that EPOM is more efficient in capturing epigenomic characteristics of different tissue/cell types. This result also shows the necessity of including Step 2 in the testing procedure to identify cell-type-specific enhancer/promoter regions.

If in Step 2 (t test) of the testing procedure we use a lower threshold *m*=13 instead of *m*=14, the discovered associated enhancers and associated promoters would become less cell-type-specific. The resulting EPOM scores are consequently less distinguishable and the correspondence maps (see Fig. 3c, d) reveal subtler similarities between different tissue and cell types. The discovered off-diagonal mappings reveal biologically meaningful relationships. For example, Heart, Muscle and Smooth Muscle are grouped together; Blood & T cells and HSC & B cells are grouped together; Neurosphere is mapped to both Brain and ES-derived cells [15]; Thymus is mapped to Blood & T cells, consistent with its role in T-cell maturation and immunity: thymus is a specialized organ of the immune system and T cells mature within thymus; Thymus is also mapped to HSC & B cells, consistent with the fact that a small population of B cells develop in thymus and some HSC colonize in thymus [16]. As the associated regions become less specific from Fig. 3a, b to Fig. 3c, d the correspondence maps based on enhancers and promoters, although present slight differences, are still consistent with each other, suggesting that our identified associated promoters and enhancers have similar levels of cell/tissue specificity in terms of grouping capability.

We also calculated the EPOM matrices for each of the two histone modification marks separately to see how different the marks’ abilities are to capture cell type characteristics. Instead of taking the union of two marks’ associated enhancers (or promoters) in Step 2, we used H3K4me1 and H3K27ac’s associated enhancers (or promoters) separately to perform the overlap test in Step 3. When using the higher threshold (*m*=14), the results from the two marks are generally the same; when using the lower threshold (*m*=13), the results from the two marks are still consistent, but with different scores for certain off-diagonal patterns (please see Additional file 3). To further study how different histone modification marks impact the EPOM scores, we added a third mark histone H3 lysine 4 tri-methylation (H3K4me3) to our study because H3K4me3 is acknowledged to be characteristic of actively transcribed protein-coding promoters [17]. We calculated EPOM scores based on associated enhancers or promoters identified from the three histone modification marks (see Additional file 1). The EPOM matrices still exhibit a strong diagonal pattern that is highly consistent with what we observed from H3K4me1 and H3K27ac.

Another case worth attention is how the EPOM scores change if we summarize the associated enhancers (or promoters) in Step 2 of the testing procedure by taking the intersection of associated enhancers (or promoters) identified for each mark (see Additional file 4). As expected, the diagonal pattern of EPOM matrices become stronger since less associated enhancers (or promoters) are shared among different tissue/cell types. But the significant off-diagonal mappings were still successfully identified.

### Potential target genes of the associated enhancers and promoters

Gene expression programs are controlled and regulated by cell-specific changes in the activity of cis-regulatory elements, including enhancers and promoters. Although identifying and annotating these regulatory elements remains a great challenge, it is possible to infer the biological functions of these regions by analyzing the functions of their neighboring genes, which are potential target genes under their regulation [18–20]. Here we study the possible functions of the identified associated enhancers and promoters by analyzing the functions of their nearby genes, which we refer to as the potential target genes of the associated enhancers and promoters.

**Fig. 4:**
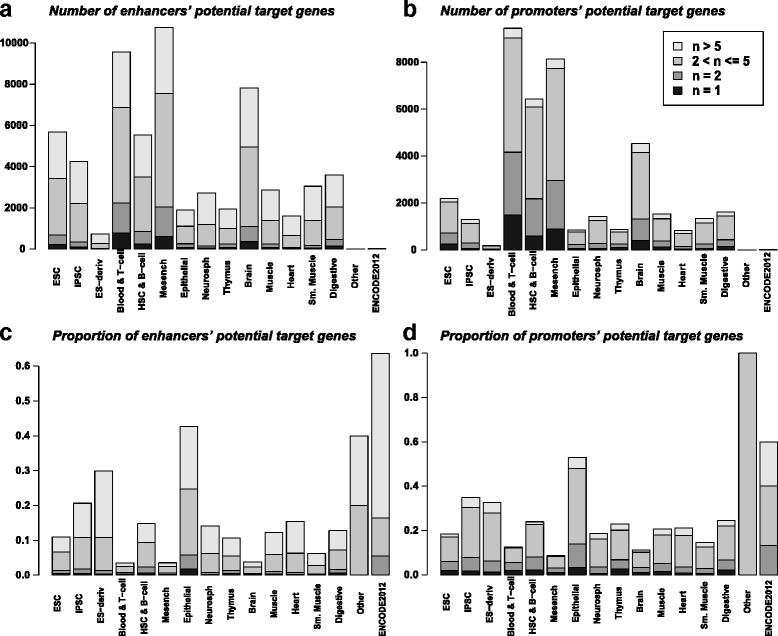
**a** Number of associated enhancers’ potential target genes in each tissue/cell type. **b** Number of associated promoters’ potential target genes in each tissue/cell type. **c**–**d** Proportions are calculated as the number of regions’ potential target genes divided by the number of associated regions. **c** Proportion of enhancers’ potential target genes in corresponding groups as in (**a**). **d** Proportion of promoters’ potential target genes in corresponding groups as in **b**. Colors of the sub-bars represent the number of tissue/cell types (*n*) in which the genes are identified, as explained in the legend

We related each associated enhancer or associated promoter to its nearest transcription start site (TSS) in up to 200 kb distance. Assignment of a gene to an associated enhancer was counted in both upstream and downstream directions, while assignment of a gene to an associated promoter was counted only in the promoter’s downstream direction. The numbers and proportions of the potential target genes assigned to the associated enhancers and promoters are summarized in Fig. 4. The distribution of the potential target gene numbers across tissue/cell types are largely consistent: more genes are identified in Blood & T-cells, HSC & B-cells, Mesenchymal stem cells, Brain and ESC. However, the target genes of associated promoters are more cell-type-specific than those of associated enhancers: larger proportion of associated promoters’ target genes are identified in unique tissue/cell types, while larger proportion of associated enhancers’ target genes are shared by more than five tissue and cell types. These results suggest that although promoters are more universal to all tissue/cell types, the associated promoters, which are non-housekeeping, are more tissue/cell type specific than the associated enhancers. The associated enhancers are more largely shared by subsets of tissue/cell types. Hence, the associated promoters are better markers of tissue/cell type specificities, while the associated enhancers are better indicators to discover tissue/cell type similarities.

Noticing that real enhancers and promoters can span across regions much longer than 200 bp, we merged the adjacent associated enhancers or promoters and re-identified the potential target genes of the merged associated enhancers or promoters (see Additional file 5). With decreasing numbers of the associated enhancers and promoters, the proportions of the target genes increase (see Fig. 4 and Additional file 5); however, the distribution of the proportions across tissue/cell types remains largely the same.

### Gene ontology enrichment analysis of associated enhancers and promoters

**Fig. 5:**
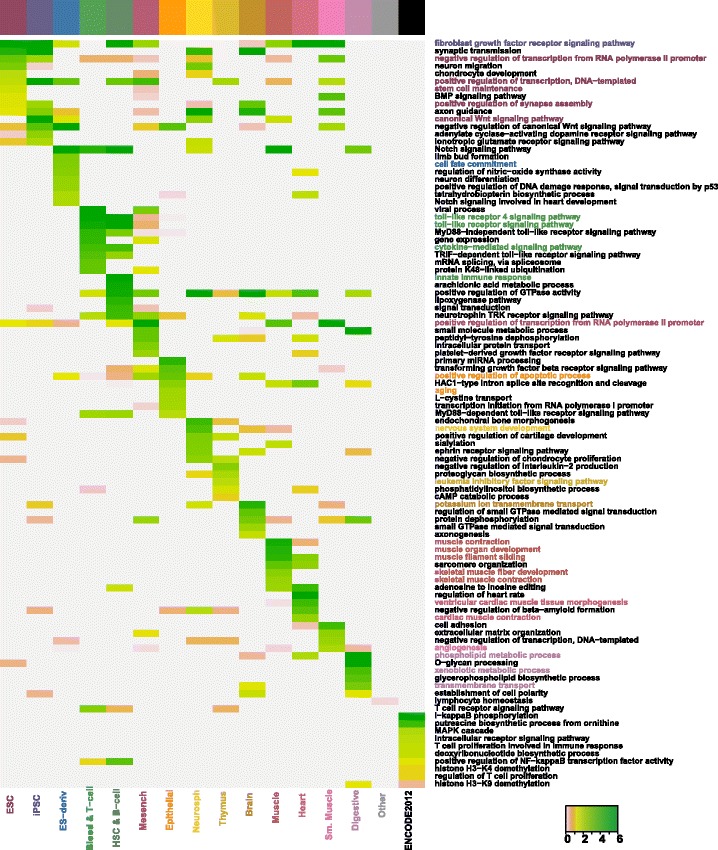
GO enrichment analysis of associated enhancers’ potential target genes. Shown GO terms are at least among the top 10 enriched GO terms in one tissue/cell type. Enrichment scores are calculated as − log10(Bonferroni corrected *p*−values). Darker colors represent higher scores. The enriched GO terms can be used to infer the biological functions of associated enhancers in each tissue/cell type. To illustrate this, some GO terms are marked in the same colors of the tissue/cell types (see the colorbar on top of the heatmap and the heatmap column labels) in which the terms are enriched

**Fig. 6:**
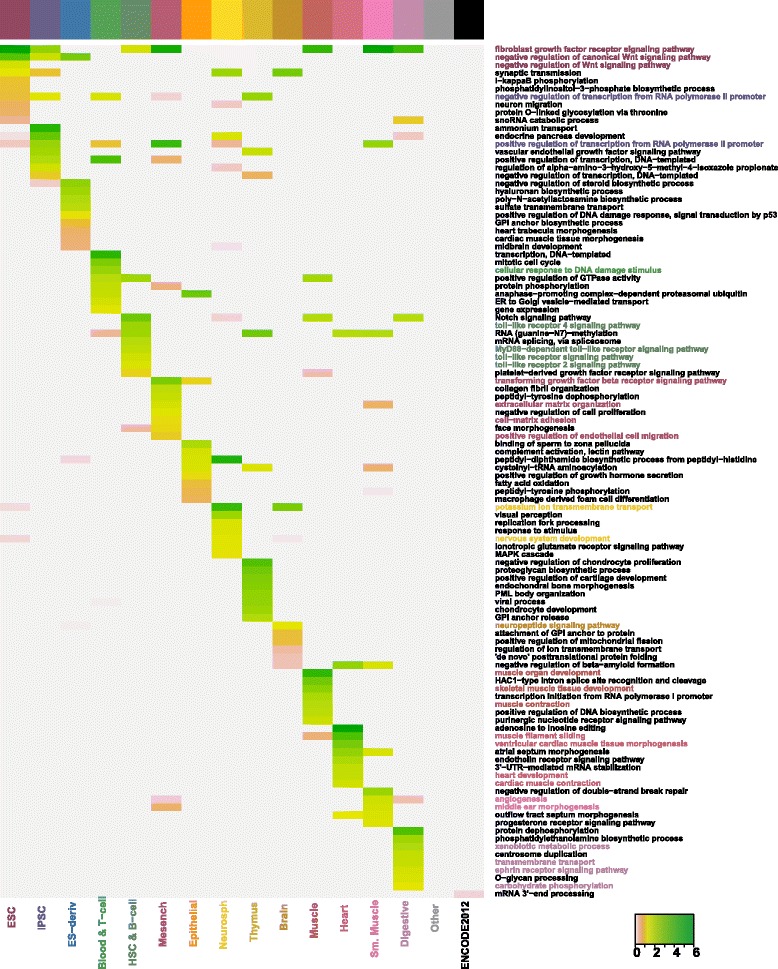
GO enrichment analysis of associated promoters’ potential target genes. Shown GO terms are at least among the top 10 enriched GO terms in one tissue/cell type. Enrichment scores are calculated as − log10(Bonferroni corrected *p*−values). Darker colors represent higher scores. The enriched GO terms can be used to infer the biological functions of associated promoters in each tissue/cell type. To illustrate this, some GO terms are marked in the same colors of the tissue/cell types (see the colorbar on top of the heatmap and the heatmap column labels) in which the terms are enriched

We performed gene ontology (GO) [21] enrichment analysis on the associated enhancers/promoters’ potential target genes of each tissue and cell type to check which GO terms are over-represented in the associated enhancers (or promoters). We used biological process GO terms and focused on the top enriched GO terms found by an overlap test in each tissue and cell type. The heatmaps of GO enrichment scores (see Figs. 5 and 6) show that the top 10 enriched GO terms in the gene lists are distinct for each tissue/cell type, with only a small proportion shared in common. We calculated the proportion of cell-type-specific GO terms–number of specifically enriched GO terms divided by number of enriched GO terms (*p*-value <10^−3^)– and found that the associated promoters have around 1.5−6 times cell-type-specific GO terms compared with the associated enhancers. This again implies that in terms of the 200 bp regions, the associated promoters are more cell-type-specific than associated enhancers.

**Fig. 7:**
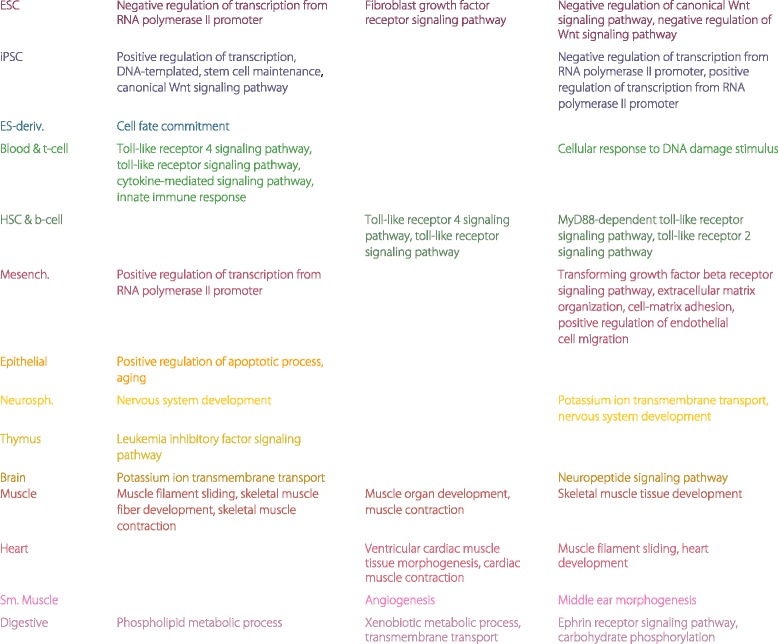
Characteristic GO terms enriched in different tissue/cell types. These terms are the characteristic ones extracted from Figs. 5 and 6. The second (fourth) column lists the GO terms only enriched in associated enhancers (promoters); the third column lists the GO terms enriched in both associated enhancers and promoters

The annotations of top enriched GO terms in each tissue and cell type (please see Additional files 6 and 7) verify and explain the similarity patterns discovered through EPOM score matrices. For instance, we observe a mapping between Heart and Muscle through the EPOM scores (Fig. 3a). Heart and Muscle actually share six common GO terms between their top 20 enriched GO terms in associated enhancers. The common GO terms include muscle filament sliding, sarcomere organization, fibroblast growth factor receptor signaling pathway, adenosine to inosine editing, positive regulation of GTPase activity and HAC1-type intron splice site recognition and cleavage (see Fig. 5). For another example, in accordance with the mapping of Blood & T-cell and HSC & B-cell in Fig. 3c, d, they share six top enriched GO terms, including toll-like receptor signaling pathway and cytokine-mediated signaling pathway. In addition, consistent with the mapping of Neurosphere and Brain, they have six top enriched GO terms in common, including synaptic transmission, positive regulation of GTPase activity and axon guidance.

**Fig. 8:**
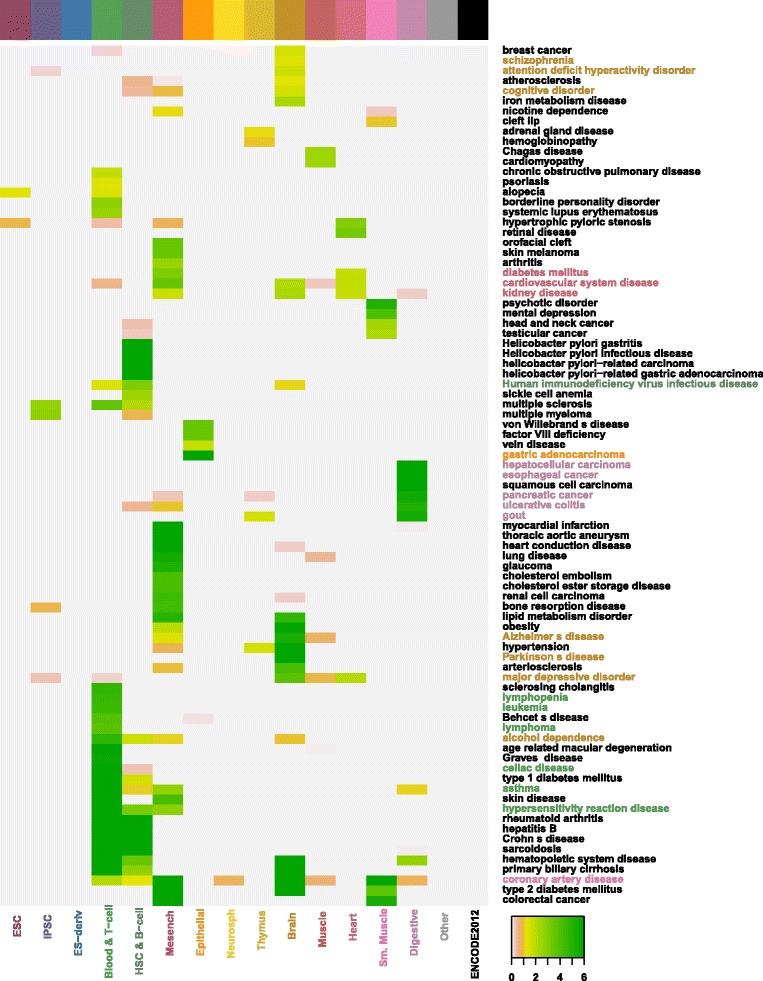
DO enrichment analysis in associated enhancers in each tissue/cell type. Shown DO terms are at least significantly enriched in one tissue/cell type. Enrichment scores are calculated as − log10(Bonferroni corrected *p*−values). Darker colors represent higher scores. For each tissue/cell type, its enriched DO term is marked in the same color as the tissue/cell label if there is a straightforward relationship between the DO term and the tissue/cell type

Many of the top enriched GO terms involve highly relevant functions of their corresponding tissue/cell types (see Figs. 5 and 6), proving that associated enhancers and promoters do carry important characteristics of the tissue and cell types. For example, it was observed that the DNA methylation pattern is very similar between iPSC and ESC but it is still possible to distinguish iPSC from ESC through differentially marked genomic regions [22]. In the GO enrichment analysis, we observe a great overlap between ESC’s and iPSC’s top enriched GO terms in associated enhancers (Fig. 5) as well as obvious distinction between ESC’s and iPSC’s top enriched GO terms in associated promoters (Fig. 6). Figure 8 provides a summary of the characteristic GO terms that are biologically relevant to each tissue/cell type. These terms serve as a good basis to understand the enhancer and promoter functions under different contexts [23]. In addition, the rest of top enriched terms imply potentially novel functions of enhancers and promoters in diverse tissue and cell types.

### GWAS and disease ontology (DO) enrichment analysis of associated enhancers and promoters

Genome-wide association studies (GWAS) have identified millions of genetic variants associated with common traits and diseases. However, selecting informative single-nucleotide polymorphisms (SNPs) that have main effects on diverse diseases remains a great challenge [24]. It was observed that many non-coding variants associated with common diseases are concentrated in regulatory sequences on human genome [25]. As a consequence, the associated enhancers and associated promoters discovered by EPOM carry important information on cell-type-specific diseases and may serve as a potential source to promote the identification of pathogenic tissue/cell types of diverse disease disorders and the understanding of regulatory mechanisms of human disease.

To verify the associated enhancers’ and promoters’ ability in capturing causal genetic variants relevant to human diseases, we first globally quantify the enrichment of trait/disease associated SNPs from GWASdb [26] in each set of associated enhancers or promoters and then carry out Disease Ontology (DO) enrichment analysis to evaluate the enriched DO terms in each tissue/cell type [27]. The global test show that trait/disease associated SNPs in the GWAS catalog are strongly enriched in the associated enhancers in most tissue/cell types while the enrichment in associated promoters is less significant. Table 3 gives the enrichment score for each tissue/cell type. The results of DO enrichment analysis match the global enrichment results: among the total 426 DO terms, 122 are enriched (-log(Bonferroni corrected *p*-values) >0) and 85 are significantly enriched (-log(Bonferroni corrected *p*-values) >1) in associated enhancers of at least one tissue/cell type; 61 are enriched and 39 are significantly enriched in associated promoters of at least one tissue/cell type (please see Fig. 8 and Additional file 8).

**Table 3:** GWAS enrichment scores

	−*l* *o* *g*(Bonferroni corrected *p*−values)	
Tissue/cell type	Associated enhancers	Associated promoters
ESC	0.00	0.00
iPSC	3.19	0.00
ES-deriv.	0.00	0.00
Blood & T-cell	89.01	32.25
HSC & B-cell	48.69	28.20
Mesench.	133.54	28.47
Epithelial	0.00	0.00
Neurosph.	2.90	0.00
Thymus	3.39	0.00
Brain	78.46	5.13
Muscle	11.47	0.00
Heart	3.36	0.00
Sm. Muscle	29.27	0.00
Digestive	6.96	3.49
Other	0.00	0.00
ENCODE2012	0.00	0.00

A series of biologically meaningful relationships between diseases and tissue/cell types are identified and verified in the enrichment analysis (please see Fig. 8 and Additional file 8). In terms of associated enhancer regions, DO terms corresponding to different hypersensitivity reaction disease (celiac disease), hematopoietic system disease (lymphopenia) and immune system cancer (lymphoma and leukemia) are enriched in Blood & T-cell and HSC & B-cell; DO terms representing hepatocellular carcinoma, pancreatic cancer and a series of gastrointestinal system disease (such as ulcerative colitis and esophageal cancer) are enriched in Digestive; DO terms representing disease of mental health (such as attention deficit hyperactivity disorder, alcohol dependence and schizophrenia), major depressive disorder and neurodegenerative disease (such as Alzheimer’s disease and Parkinson’s disease) are enriched in Brain; Cardiovascular system disease is enriched in both Muscle and Heart; and gastric adenocarcinoma (which derives from epithelial cells of glandular origin) is enriched in Epithelial. In terms of associated promoter regions, similar diseases as in associated enhancers were found to be enriched in Blood & T-cell, HSC & B-cell, Digestive and Epithelial. In addition, type 1 diabetes mellitus is also enriched in Digestive and cardiomyopathy (characterized by deterioration of the function of the heart muscle) is enriched in Muscle. Moreover, some more complicated relationships between diseases and tissue/cell types are also recovered in the DO enrichment analysis. For example, diabetes mellitus and kidney disease are found to be enriched in Heart while research have shown that both diabetes and kidney disease are high risk factors for heart disease [28, 29].

## Discussion and conclusions

In this work, we propose a new measure for comparing and grouping biological samples from different tissue and cell types: Epigenomic Overlap Measure (EPOM). EPOM compares different tissue and cell types based on the similarity of histone modification marks evaluated in their relevant chromatin states. The proposed measure is calculated via a three-step testing procedure including ANOVA, t test and overlap test. Compared to traditional correlation analysis, EPOM is able to create a much clearer mapping pattern across 16 tissue and cell types. By tuning the thresholds in the testing procedure, EPOM can perform either grouping or identity mapping of biological samples based on epigenomic features. The associated enhancers and associated promoters identified by EPOM are good indicators of tissue/cell epigenomic characteristics, and they are important genomic regions for downstream analysis such as regulatory network analysis, GO enrichment analysis and GWAS studies. Results under different settings (i.e., by taking union or intersection of the associated regions identified for different marks; by using two or three HMs together or separately using individual marks; by using 200 bp associated regions or merged longer associated regions) all demonstrate the effectiveness of our approach compared with correlation analysis in finding clear correspondence maps of biological samples. Moreover, the resulting EPOM scores reveal biologically meaningful patterns between similar tissue/cell types and confirm the belief that epigenomic landscapes are powerful resources for understanding cellular identity [30, 31]. These results imply the great potential of using EPOM to study tumor heterogeneity based on single-cell epigenomic data [32].

The EPOM method can be easily extended to study the relationships between diverse tissue/cell types based on signals of any epigenetic marks in genomic regions of interest. Here we suggest an efficient approach to systematically select epigenetic marks for EPOM if no specific marks are of prior interest. The selection will be based on the number of regions where each mark has differential signals across biological conditions. The differential regions of each mark can be found by the Step 1 (ANOVA) in our testing procedure given a specified *p*-value threshold, and the marks that have large numbers of differential regions will be good candidates for EPOM. The rationale behind this selection approach is that EPOM prefers the marks carrying more cell-type-specific information on the genomic regions of interest. We implement this selection approach in Additional file 9, which shows that among the eight epigenetic marks studied by the Roadmap Consortium, the three marks H3K4me1, H3K27ac and H3K4me3 we use in this work are among the top ones in terms of the numbers of differential enhancer and promoter regions.

We identified the associated enhancers/promoters’ potential target genes in each tissue and cell type and used the top enriched GO terms in these genes to predict the biological functions of the associated enhancers and promoters. The results of GO enrichment analysis confirm the similarities of tissues and cell types found by EPOM and provide functional explanations for the underlying regulatory mechanisms leading to these patterns. The EPOM scores, together with the GO enrichment results, suggest that the associated enhancers and promoters have well captured the epigenomic characteristics of their corresponding tissue and cell types. An important future direction is to incorporate three-dimensional (3D) chromatin structures into the identification of the target genes of associated enhancers/promoters. The Hi-C technology makes it possible to decipher 3D chromatin structures and to thus reveal more accurate and complete interactions between genes and regulatory regions [33, 34]. However, Hi-C data are not yet available for the human tissue and cell types in our study, and without the data it is difficult to accurately infer potential target genes of associated enhancers/promoters from 3D chromatin structures [35]. In addition, better computational tools are needed for accurate 3D genome reconstruction from Hi-C data [36].

Despite the previous belief that chromatin states at promoters are largely invariant across diverse cell types [9, 37], our functional analyses on the potential target genes of the associated promoters in different tissue/cell types suggest that the non-housekeeping promoters carry cell-type-specific functions. We also found that the potential target genes of the associated enhancers are enriched with functions both specific to a single tissue/cell type or shared by a subgroup of tissue/cell types. Those associated regulatory regions identified by EPOM are key elements for understanding differential gene expression, cell differentiation and phenotypic variations.

More functional analyses based on disease ontology further confirm that the discovered associated regions carry important disease-relevant characteristics of their corresponding tissue/cell types. The identified associated enhancers and promoters can be good resources for understanding the epigenomic mechanisms of different tissue and cell types. It is a great challenge now to interpret the biological mechanisms and effects of the large amounts of identified SNPs. A common approach was to simply study the overlap between the SNPs and regulatory elements such as histone modification marks, binding sites of transcription factors and promoter regions [38]. However given that the dynamics of trait-associated variants can vary significantly in different tissue and cell types, we should carefully evaluate the enrichment of trait-associated variations in their most relevant tissues or cell types [39]. With the knowledge that our associated enhancers and promoters carry significant regulatory epigenomic features and thus represent the genomic context of their corresponding tissue and cell types better than other non-coding genomic regions, we highlight three important perspectives to make use of associated enhancers and promoters in GWAS studies. First, the identified associated enhancers and promoters provide a unique source for studying cell-type-specific disease variants and exploring disease-associated SNP functions. Although previous research showed SNP and GWAS enrichment in diverse chromatin states [12] and studied SNPs for certain selected traits [40], they did not provide a method to test the enrichment of genome-wide SNPs in cellular specific contexts. Second, the enriched DO terms can help researchers understand the dynamics of disease-related regulatory elements across diverse tissue/cell types. We can identify the potential target genes of the associated enhancers and promoters highly enriched with disease-related SNPs. Then by comparing the distinct and common target genes of each tissue/cell type and studying the regulatory networks between those genes and their associated enhancers or promoters, it is possible to shed light on the causes of cell type specific diseases as well as multi-factorial disorders. Last, the results of our study provide useful information to refine the disease ontology. Once we verify the potential target genes of the associated enhancers (or promoters) enriched with disease variants, we can update the DO terms to reflect these newly discerned genes [41].

## Availability of supporting data

The epigenomic datasets supporting the results of this article are available at the web portal of the Roadmap Epigenomics Project. Both the data of the 25-state Imputation Based Chromatin State Model and the imputed signals of histone modification marks are available at http://egg2.wustl.edu/roadmap/web_portal/imputed.html#chr_imp. The data for SNP annotation is available at http://jjwanglab.org/gwasdb. The associated enhancer and promoter regions identified by EPOM are available at http://www.stat.ucla.edu/~jingyi.li/software-and-data.html or http://www.stat.ucla.edu/~jingyi.li/data/EpOM/associated_enhancers_and_promoters.tar.gz.zip.

## Additional files

Additional file 1
**Figure S1.** Correspondence maps of EPOM scores saturated at 20. The associated enhancers (promoters) used are the unions of the associated enhancers (promoters) identified through each histone modification mark (H3K4me1, H3K27ac and H3K4me3) in step 2. (PDF 420 kb)

Additional file 2
**Figure S2.** Correspondence maps of Pearson correlation coefficients calculated from H3K4me1 and H3K27ac on candidate associated enhancers and candidate associated promoters after step 1 (ANOVA). (PDF 2334 kb)

Additional file 3
**Figure S3.** Correspondence maps of EPOM scores saturated at 20. Each heatmap plots the EPOM scores calculated from associated regions (enhancers or promoters) identified through one histone modification mark (H3K4me1 or H3K27ac) in step 2. (PDF 434 kb)

Additional file 4
**Figure S4.** Correspondence maps of EPOM scores saturated at 20. The associated enhancers (promoters) used to calculate EPOM scores are the intersection of the the associated enhancers (promoters) identified for the two histone modification marks. (PDF 416 kb)

Additional file 5
**Figure S5.** Numbers and proportions of potential target genes identified for merged associated enhancers and merged associated promoters. (PDF 493 kb)

Additional file 6
**Table S1.** Top 20 enriched GO terms in enhancer-associated genes in each tissue/cell type. (XLSX 15 kb)

Additional file 7
**Table S2.** Top 20 enriched GO terms in promoter-associated genes in each tissue/cell type. (XLSX 16 kb)

Additional file 8
**Figure S6.** DO enrichment analysis on associated promoter regions in each tissue/cell type. (PDF 169 kb)

Additional file 9
**Table S3.** Number of candidate associated enhancers (or promoters) of the eight histone modifications marks. (XLSX 10 kb)
