# Supplementary material for: Epigenome overlap measure (EPOM) for comparing tissue/cell types based on chromatin states
Source: BMC Genomics. 2016 Jan 11;17(Suppl 1):10. doi: 10.1186/s12864-015-2303-9 (PMC4895267; doi:10.1186/s12864-015-2303-9)
Supplement: Additional file 1 — Figure S1. Correspondence maps of EPOM scores saturated at 20. The associated enhancers (promoters) used are the unions of the associated enhancers (promoters) identified through each histone modification mark (H3K4me1, H3K27ac and H3K4me3) in step 2. (PDF 420 kb) [file 12864_2015_2303_MOESM1_ESM.pdf]

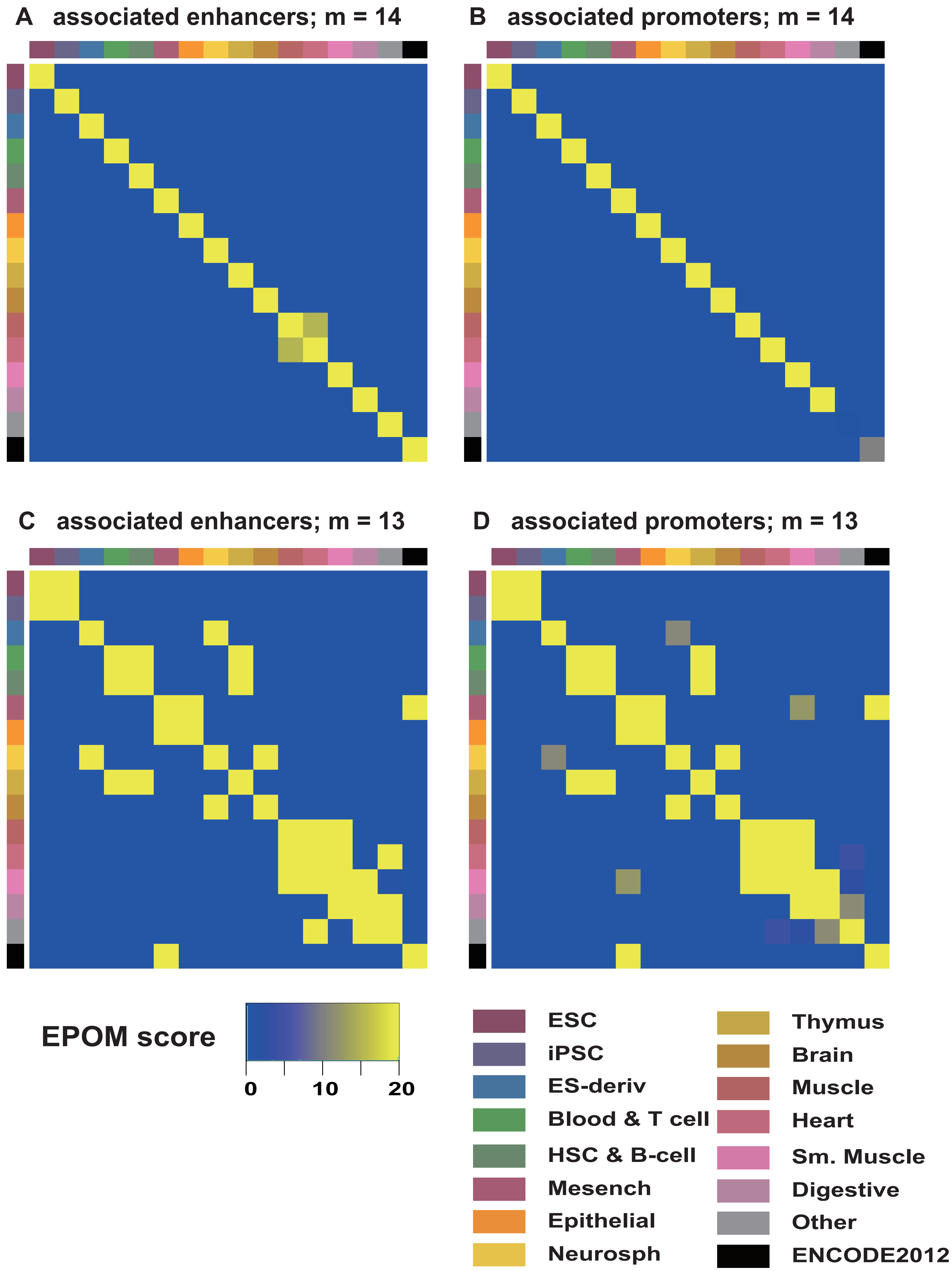

Figure S1. Correspondence maps of EPOM scores saturated at 20. The associated enhancers (promoters) used to calculate EPOM scores are the unions of the associated enhancers (promoters) identified through each histone modification mark (H3K4me1, H3K27ac and H3K4me3) in step 2. A-B: theshold m=14 in step 3. C-D: threshold m=13 in step 3.
