# Supplementary material for: Epigenome overlap measure (EPOM) for comparing tissue/cell types based on chromatin states
Source: BMC Genomics. 2016 Jan 11;17(Suppl 1):10. doi: 10.1186/s12864-015-2303-9 (PMC4895267; doi:10.1186/s12864-015-2303-9)
Supplement: Additional file 2 — Figure S2. Correspondence maps of Pearson correlation coefficients calculated from H3K4me1 and H3K27ac on candidate associated enhancers and candidate associated promoters after step 1 (ANOVA). (PDF 2334 kb) [file 12864_2015_2303_MOESM2_ESM.pdf]

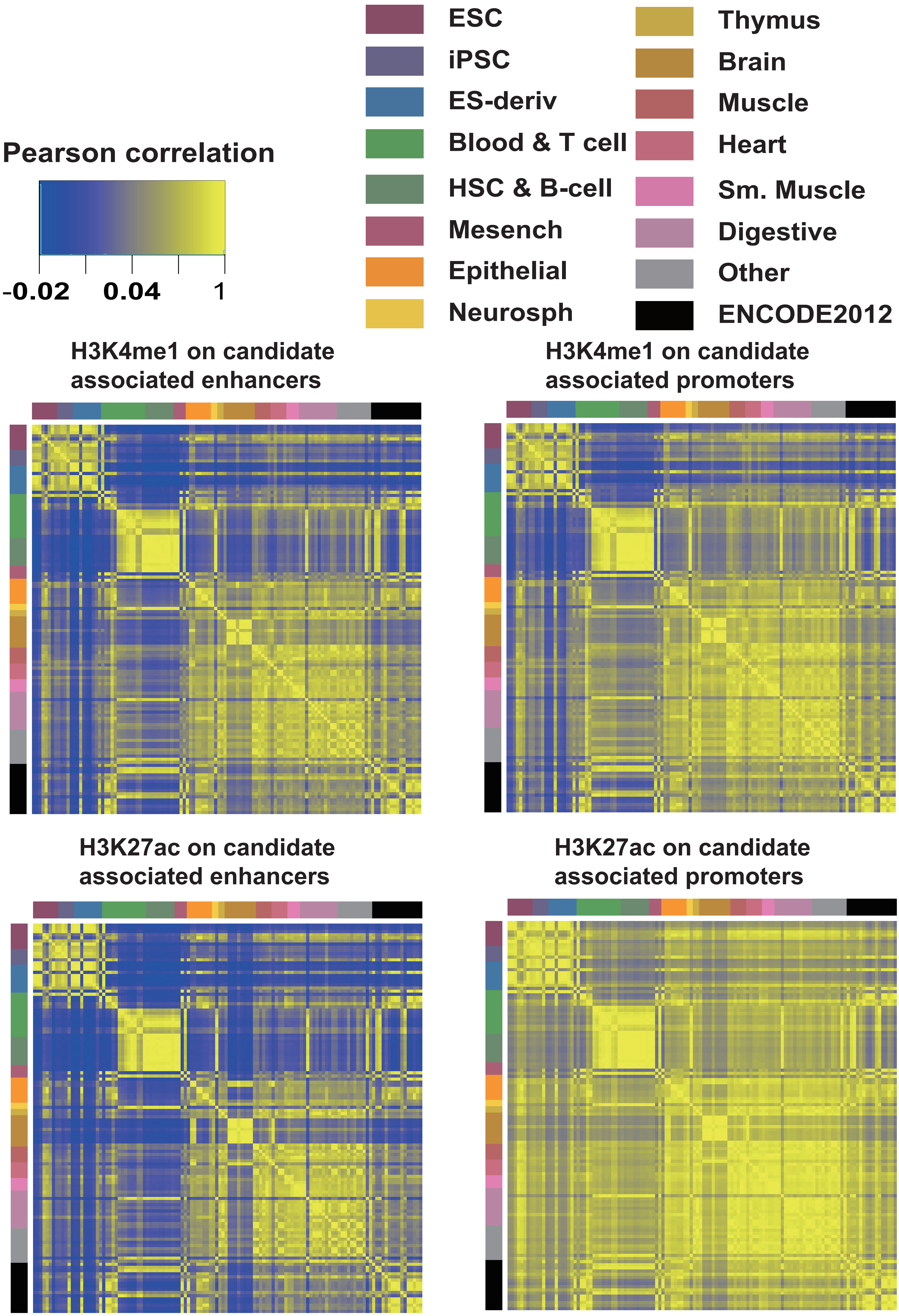

Figure S2. Correspondence map of Pearson correlation coefficients calculated from H3K4me1 and H3k27ac on candidate associated enhancers and candidate associated promoters after step 1 (ANOVA).
