# Supplementary material for: Epigenome overlap measure (EPOM) for comparing tissue/cell types based on chromatin states
Source: BMC Genomics. 2016 Jan 11;17(Suppl 1):10. doi: 10.1186/s12864-015-2303-9 (PMC4895267; doi:10.1186/s12864-015-2303-9)
Supplement: Additional file 3 — Figure S3. Correspondence maps of EPOM scores saturated at 20. Each heatmap plots the EPOM scores calculated from associated regions (enhancers or promoters) identified through one histone modification mark (H3K4me1 or H3K27ac) in step 2. (PDF 434 kb) [file 12864_2015_2303_MOESM3_ESM.pdf]

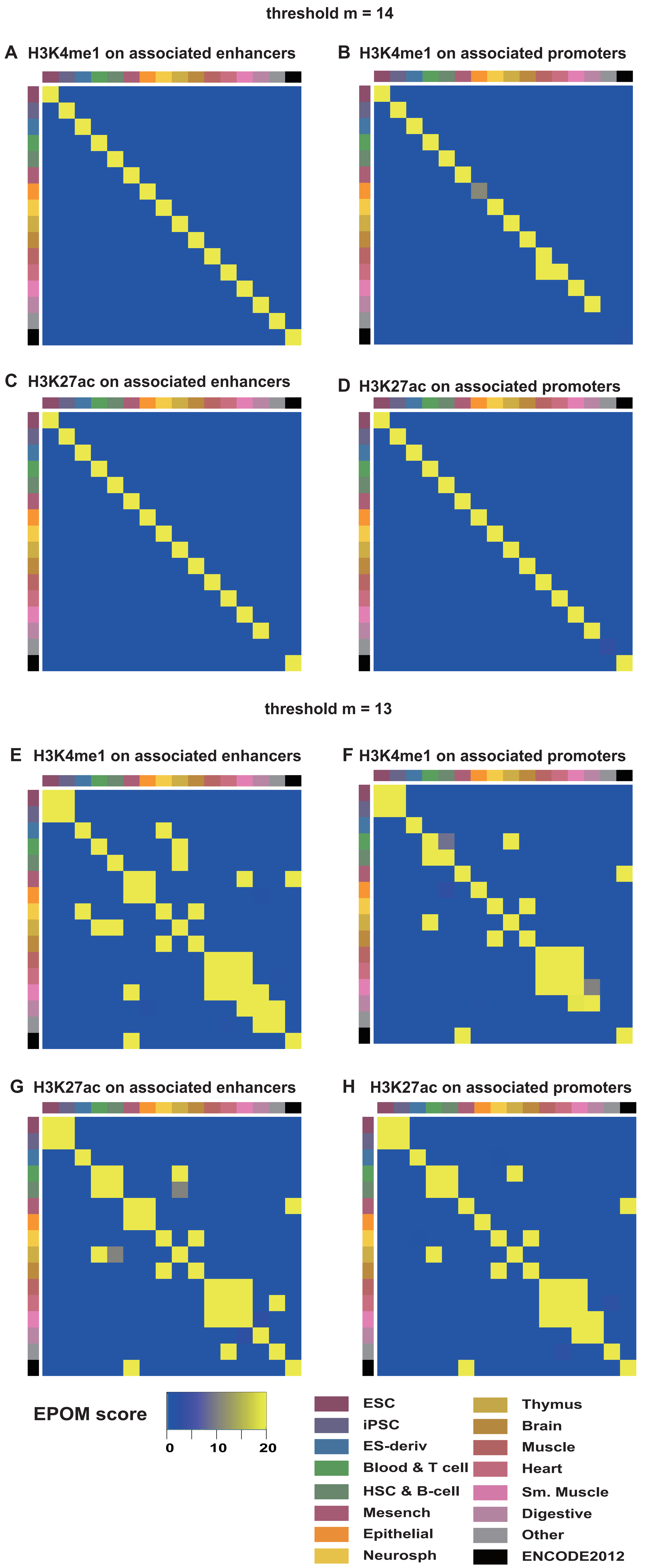

Figure S3. Correspondence maps of EPOM scores saturated at 20. Each heatmap plots the EPOM scores calculated from associated regions (enhancers or promoters) identified through one histone modification mark (H3K4me1 or H3K27ac) in step 2. A-D: theshold m=14 in step 3. E-H: threshold m=13 in step 3.
