# Supplementary material for: Epigenome overlap measure (EPOM) for comparing tissue/cell types based on chromatin states
Source: BMC Genomics. 2016 Jan 11;17(Suppl 1):10. doi: 10.1186/s12864-015-2303-9 (PMC4895267; doi:10.1186/s12864-015-2303-9)
Supplement: Additional file 4 — Figure S4. Correspondence maps of EPOM scores saturated at 20. The associated enhancers (promoters) used to calculate EPOM scores are the intersection of the the associated enhancers (promoters) identified for the two histone modification marks. (PDF 416 kb) [file 12864_2015_2303_MOESM4_ESM.pdf]

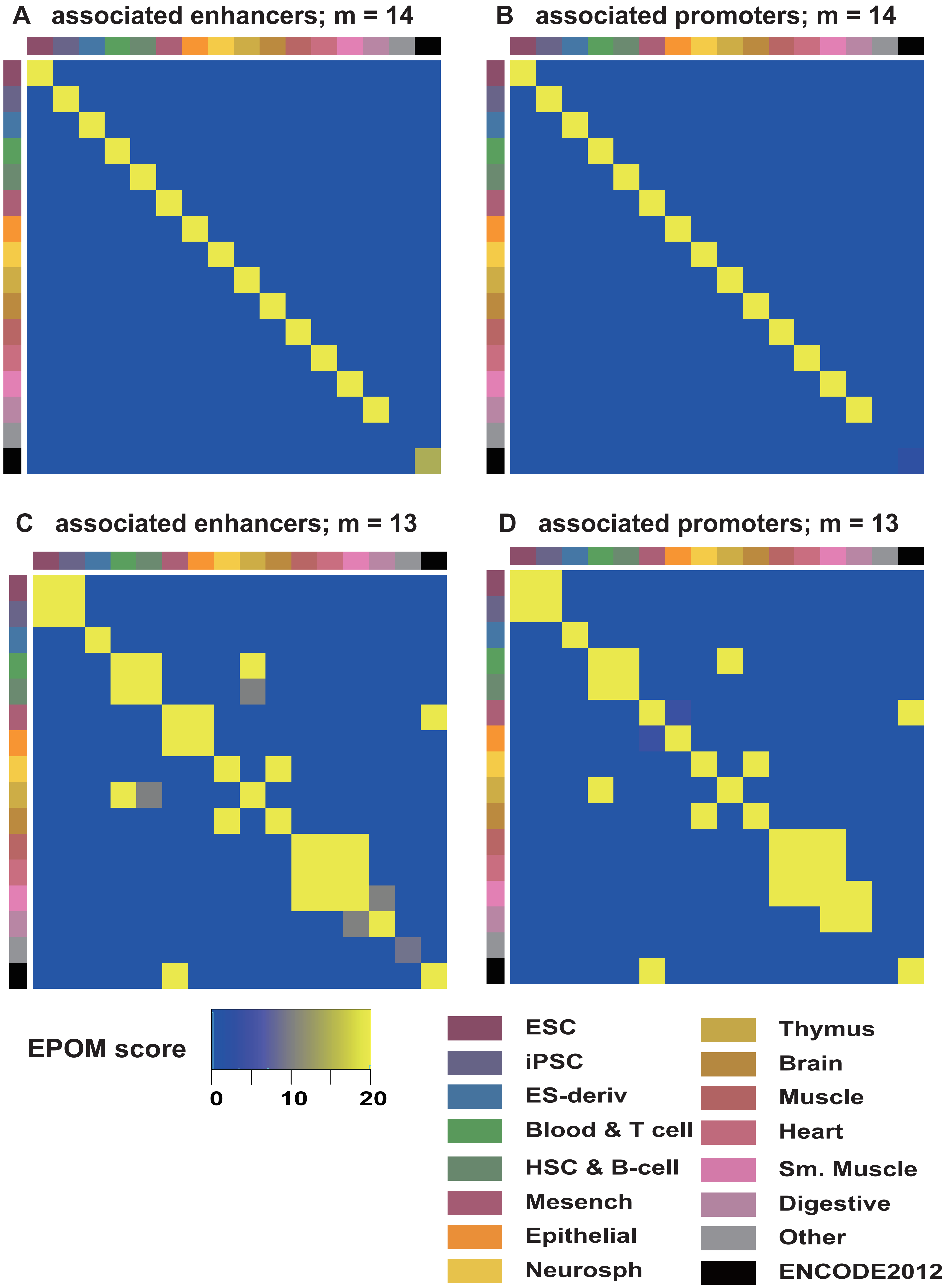

Figure S2. Correspondence maps of EPOM scores saturated at 20. The associated enhancers (promoters) used to calculate EPOM scores are the intersection of the the associated enhancers (promoters) identified for the two histone modification marks. A-B: theshold m=14 in step 3. C-D: threshold m=13 in step 3.
