# Supplementary material for: Epigenome overlap measure (EPOM) for comparing tissue/cell types based on chromatin states
Source: BMC Genomics. 2016 Jan 11;17(Suppl 1):10. doi: 10.1186/s12864-015-2303-9 (PMC4895267; doi:10.1186/s12864-015-2303-9)
Supplement: Additional file 5 — Figure S5. Numbers and proportions of potential target genes identified for merged associated enhancers and merged associated promoters. (PDF 493 kb) [file 12864_2015_2303_MOESM5_ESM.pdf]

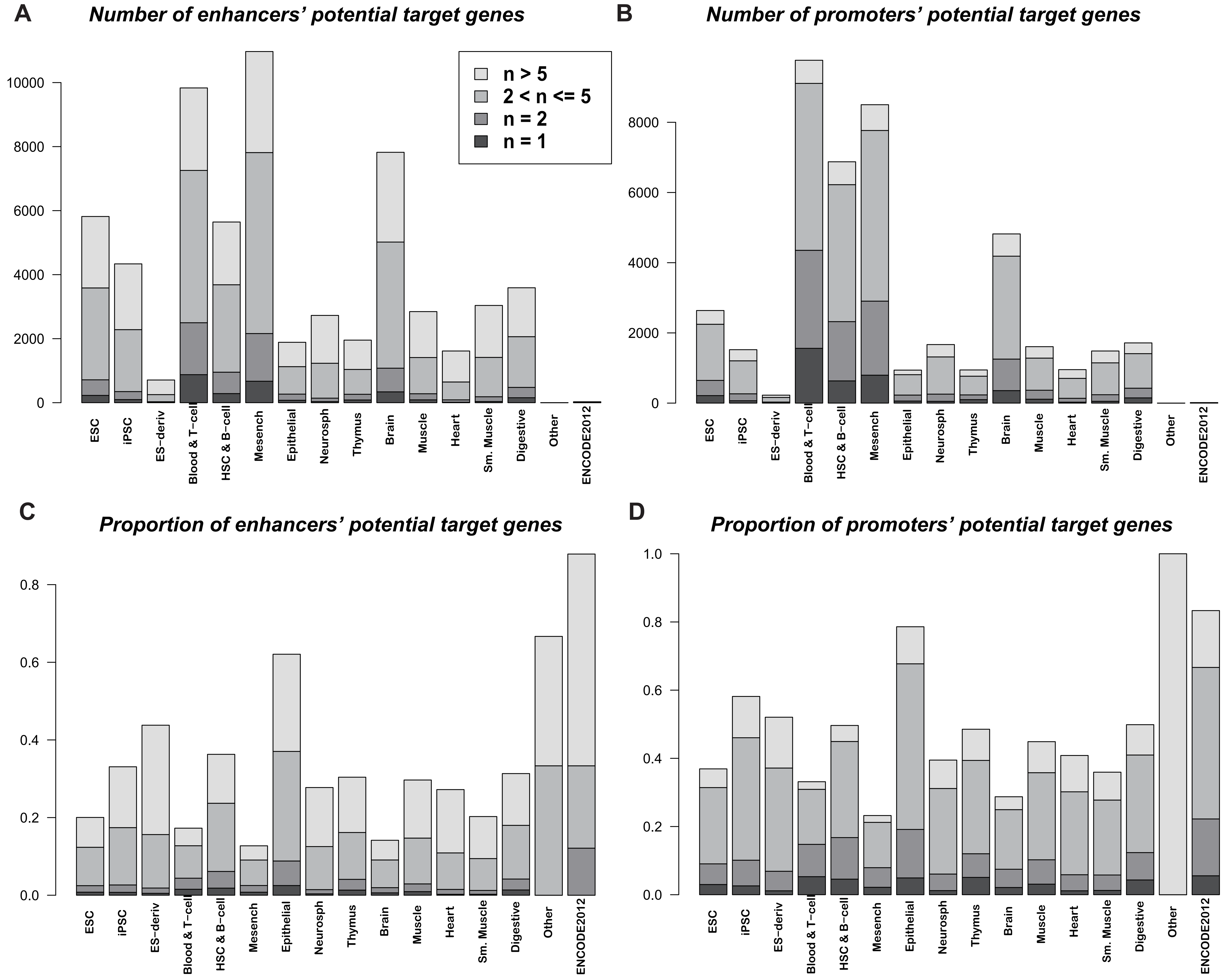

Figure S3. Numbers and proportions of enhancers/promoters' potential target genes. Adjacent 200-bp regions of associated enhancers/promoters were merged into a single longer region and taken as new associated enhancers and associated promoters. Potential target genes were identified for the new associated enhancers and promoters after merging. A: Number of enhancers' potential target genes in each tissue/cell type. B: Number of promoters' potential target genes in each tissue/cell type. C-D: Proportions are counted as the number of regions' potential target genes divided by the number of associated regions. C: Proportion of enhancers' potential target genes in corresponding groups as in A. D: Proportion of penhancers' potential target genes in corresponding groups as in B. Colors of the sub-bars represent the number of tissue/cell types (n) in which the genes are identified, as explained in the legend.
