# Supplementary material for: Epigenome overlap measure (EPOM) for comparing tissue/cell types based on chromatin states
Source: BMC Genomics. 2016 Jan 11;17(Suppl 1):10. doi: 10.1186/s12864-015-2303-9 (PMC4895267; doi:10.1186/s12864-015-2303-9)
Supplement: Additional file 8 — Figure S6. DO enrichment analysis on associated promoter regions in each tissue/cell type. (PDF 169 kb) [file 12864_2015_2303_MOESM8_ESM.pdf]

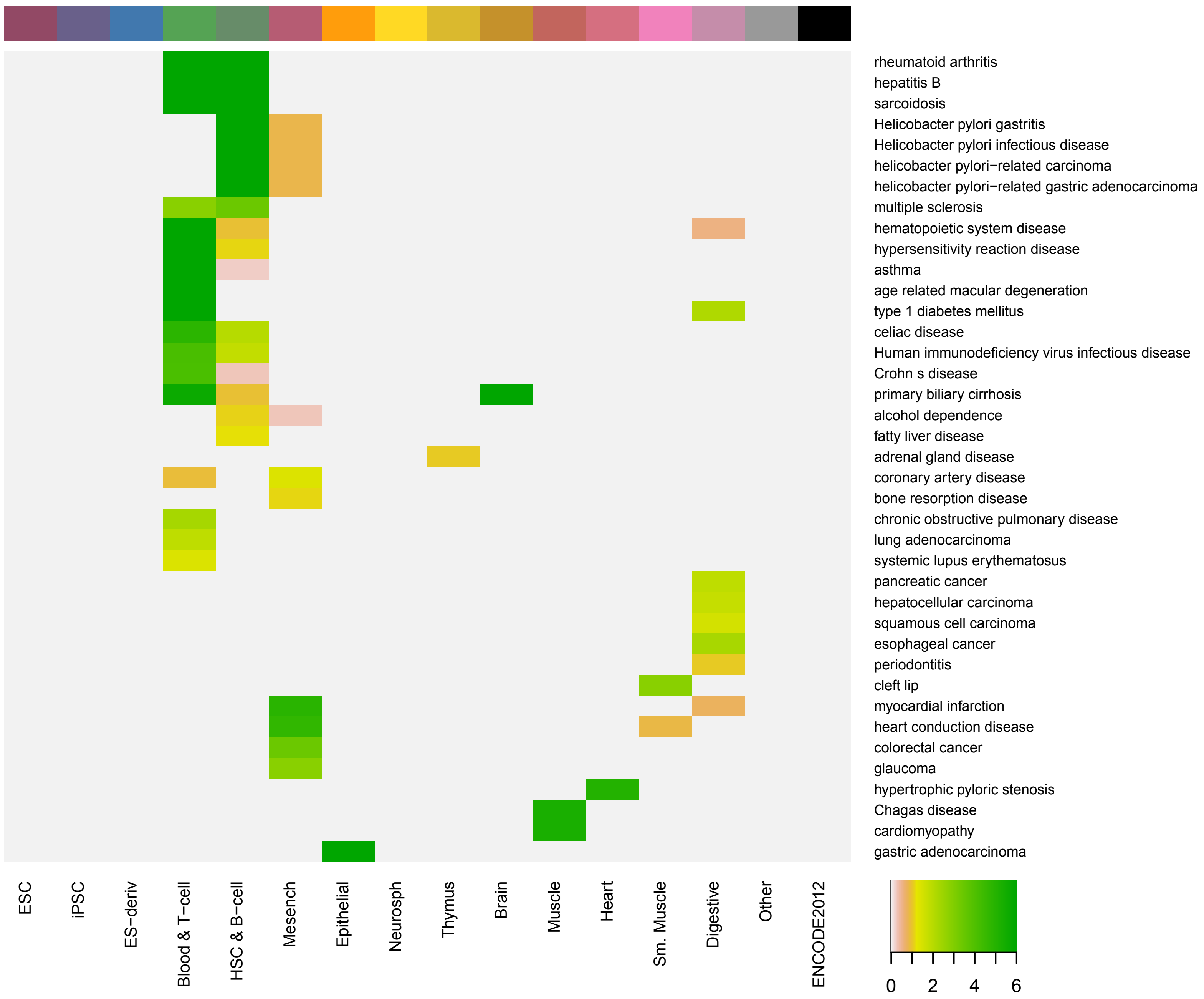

Figure S6. DO enrichment analysis on associated promoter regions in each tissue/cell type. Shown DO terms are at least significantly enriched in one tissue/cell type. Enrichment scores are calculated as -log_10(Bonferroni corrected p-values). Darker colors represent higher scores.
